# Review of *Mesocallis* Matsumura from China (Hemiptera, Aphididae), with one new species

**DOI:** 10.3897/zookeys.1003.56563

**Published:** 2020-12-14

**Authors:** Jing Chen, Li-Yun Jiang, Ge-Xia Qiao

**Affiliations:** 1 Key Laboratory of Zoological Systematics and Evolution, Institute of Zoology, Chinese Academy of Sciences, No. 1-5 Beichen West Road, Chaoyang District, Beijing 100101, China Institute of Zoology, Chinese Academy of Sciences Beijing China; 2 College of Life Science, University of Chinese Academy of Sciences, No. 19, Yuquan Road, Shijingshan District, Beijing 100049, China University of Chinese Academy of Sciences Beijing China

**Keywords:** Aphids, Calaphidinae, embryo, new host plant

## Abstract

The aphid genus *Mesocallis* Matsumura in China is reviewed. A total of seven species are recognised using morphological characteristics, including six known species, Mesocallis (Mesocallis) alnicola Ghosh, M. (Paratinocallis) corylicola (Higuchi), M. (M.) pteleae Matsumura, M. (M.) sawashibae (Matsumura), M. (P.) yunnanensis (Zhang) and M. (M.) taoi Quednau, and one new species, M. (M.) platycaryae Qiao, **sp. nov.** The new species, collected on *Platycarya strobilacea* (Juglandaceae) in Anhui Province, China, is described and illustrated. A key to *Mesocallis* species from China is presented.

## Introduction

The aphid genus *Mesocallis* was erected by Matsumura (1919), with *Myzocallis sawashibae* Matsumura, 1917 as the type species. The genus has distinct morphological characteristics; a narrow body, antennae much shorter than the body, antennal segments IV–VI scarcely imbricated, segment III of the alatae with one row of oblong, secondary rhinaria along all or most of its length, and empodial setae distinctly longer than the claws (Higuchi 1972; Quednau 2003; Qiao et al. 2005). Currently, this genus includes 10 species placed in two subgenera: Mesocallis (Mesocallis) alnicola Ghosh, M. (Paratinocallis) corylicola (Higuchi), M. (M.) carpinicola Lee, M. (M.) fagicola Matsumura, M. (M.) obtusirostris Ghosh, M. (P.) occulta Lee, M. (M.) pteleae Matsumura, M. (M.) sawashibae (Matsumura), M. (P.) yunnanensis (Zhang), and *M. taoi* Quednau (Blackman and Eastop 2020; Favret 2020). These species all are associated with plants of the family Betulaceae and are mainly distributed in East Asia. Six species are hitherto recorded from China: *M. alnicola*, *M. corylicola*, *M. pteleae*, *M. sawashibae*, *M. taoi*, and *M. yunnanensis*. Recently, some apterous specimens on *Platycarya strobilacea* (Juglandaceae) were collected in Anhui Province (Dabieshan Mountain), which were identified as a new species in this genus. Herein, the genus *Mesocallis* from China is reviewed, a key to Chinese species is provided, and the new species is described and illustrated.

## Materials and methods

The brief procedure of making aphid slide-mounted specimens follows that of Jiang et al. (2016). The descriptions and drawings provided here were produced from slide-mounted specimens using a Leica DM4000B and drawing tube. The photomicrographs were prepared with a Leica DM2500 using DIC illumination and processed with Automontage and Photoshop software.

Aphid terminology in this paper generally follows that of Quednau (2003) and Qiao et al. (2005). The unit of measurements is millimetres (mm). All specimens, including the holotype and paratypes, are deposited in the National Zoological Museum of China, Institute of Zoology, Chinese Academy of Sciences, Beijing, China (**NZMC**).

## Taxonomy

### Mesocallis

Taxon classificationAnimaliaHemipteraAphididae

Matsumura, 1919

0CF0B5E0-C0B3-5628-A01B-D14CA1073228

Mesocallis Matsumura 1919: 103. Type species: Myzocallis sawashibae Matsumura, 1917; by original designation.
Subgenus MesocallisMatsumura 1919: 103
Synonym: NeocallisMatsumura 1919: 104.
Synonym: NippochaitophorusTakahashi 1961: 247.
Subgenus ParatinocallisHiguchi 1972: 30. Type species: Paratinocallis corylicola Higuchi,1972; by original designation. Given subgenus status by Quednau (2003: 20).
Mesocallis Matsumura: Higuchi 1972: 22; Raychaudhuri et al. 1980: 291; Ghosh and Quednau 1990: 114; Quednau 2003: 19; Qiao et al. 2005: 194; Lee et al. 2018: 3.

#### Generic diagnosis.

In alatae, eyes with ocular tubercles. Antennae 6-segmented, processus terminalis 0.80–1.20× as long as the base of the segment. Ultimate rostral segment with 2–16 accessory setae. First tarsal segments with five to seven ventral setae and two dorsal setae. Empodial setae flabellate. Siphunculi truncated, without flange, without any setae surrounding at base. Cauda knobbed. Anal plate bilobed. Gonapophyses fused, with eight gonosetae. In apterae, nymphs and embryo, dorsal body setae with spinulose shafts, and round knobs at apex. Abdominal tergites I–IV with one pair of marginal setae in subgenus Mesocallis, or two or three pairs of marginal setae in subgenus Paratinocallis. Compound eye of apterous morph often smaller and with fewer facets than in the alate morph, and inner setae of antennal segment III inconspicuous. Apterae with 5- or 6-segmented antennae, dorsal setae of tibiae similar to other tibial setae in subgenus Mesocallis, or strongly differentiated from other tibial setae in subgenus Paratinocallis; first tarsal segments with five ventral setae, without dorsal setae. In embryo, dorsal body setae capitate at apex; spinal setae of metanotum and tergites I, III, and V short or minute, pleural setae absent. Viviparae alate and apterous in some species.

#### Distribution.

China, Japan, Korea, and India.

#### Host plants.

*Alnus*, *Carpinus*, *Corylus*, and *Ostrya* (Betulaceae), and *Platycarya* (Juglandaceae).

#### Comments.

Of the known *Myzocallis* species, most infest plants of Betulaceae. Two species (*obtusirostris* and *taoi*) are primarily associated with *Alnus*; and three species (*corylicola*, *occulta*, and *yunnanensis*) are associated with *Corylus*. *Myzocallis carpinicola* is recorded only on *Carpinus*. Additionally, *M. alnicola* infests both *Alnus* and *Corylus*, and *M. sawashibae* occurs on both *Carpinus* and *Corylus* (Lee et al. 2018). Only *M. pteleae* infests plants in several different genera of Betulaceae (*Alnus*, *Betula*, *Corylus*, *Carpinus*, and *Ostrya*) (Holman 2009). So, *Mesocallis* species have distinct host specialization. However, the new species, *M. platycaryae* Qiao was found on *Platycarya* (Juglandaceae). All species occur only in East Asia and are endemic to this region.

### Mesocallis (Mesocallis) alnicola

Taxon classificationAnimaliaHemipteraAphididae

Ghosh, 1974

012985EA-4157-5DCE-A69A-C5B13E5DC398

Mesocallis alnicola Ghosh 1974: 425.
Mesocallis alnicola Ghosh: Raychaudhuri et al. 1980: 292; Ghosh and Quednau 1990: 116; Quednau 2003: 20; Qiao et al. 2005: 195.

#### Specimens examined.

Two alate viviparous femlaes, **China**: Gansu (Yuzhong County: Xinglong Mountain, alt. 2300 m), 1 Aug. 1986, no. 8579, on *Corylus heterophylla*, coll. G.X. Zhang, J.H. Li, and T.S. Zhong (NZMC).

#### Distribution.

China (Gansu), India.

#### Host plants.

*Corylus heterophylla* in China (first record from this host), *Alnus nepalensis* in India.

#### Biology.

Yellow in life. Infesting the underside of leaves of host plants.

### Mesocallis (Paratinocallis) corylicola

Taxon classificationAnimaliaHemipteraAphididae

(Higuchi, 1972)

8E765B45-FF42-50B1-B703-D0A46A81653B

Paratinocallis corylicola Higuchi 1972: 30; Qiao et al. 2005: 210.
Mesocallis (Paratinocallis) corylicola (Higuchi): Quednau 2003: 21; Lee et al. 2018: 8.

#### Specimens examined.

Four alate viviparous females and 2 nymphs, **China**: Heilongjiang (Harbin City), 27 Jul. 1976, no. 6423, on *Corylus heterophylla*, coll. G.X. Zhang and T.S. Zhong (NZMC); 3 alate viviparous females, Liaoning (Shenyang City), 25 May 1984, no. Y4994, on *Corylus heterophylla*, coll. L.J. Liu and Y.Q. Wang (NZMC); 2 alate viviparous females, Liaoning (Dandong City), 22 Jun. 1984, no. Y4914, on *Corylus heterophylla*, coll. G.X. Zhang and L.J. Liu (NZMC); 7 alate viviparous females, Shandong (Taian City), 12 Jun. 1975, no. 5990, on *Corylus heterophylla*, coll. T.S. Zhong (NZMC); 2 alate viviparous females, Gansu (Yuzhong County: Xinglong Mountain, alt. 2170 m), 30 Jul. 1986, no. 8556, on *Corylus heterophylla*, coll. G.X. Zhang, J.H. Li, and T.S. Zhong (NZMC).

#### Distribution.

China (Liaoning, Heilongjiang, Shandong, Gansu), Japan, Korea.

#### Host plants.

*Corylus sieboldiana* and *C. heterophylla*; however, in the Russian Far East it was collected from *Quercus dentata* (Holman 2009).

#### Biology.

Beige or pale green in life; scattered on the underside of leaves of host plants.

### Mesocallis (Mesocallis) platycaryae

Taxon classificationAnimaliaHemipteraAphididae

Qiao sp. nov.

C3862431-1DC9-52C2-BAC0-8B2648BC2384

http://zoobank.org/F0D1DBB8-684E-4983-8CA1-47EF630AFC21

Figures 1–12 , 13–15 , 16–24 , Table 1

#### Etymology.

The specific name *platycaryae* is based on the host plant (*Platycarya*) of the species.

#### Description.

Apterous viviparous female: body oval (Fig. 13), translucent white in life. For morphometric data, see Table 1.

**Table 1.** Morphometric data for apterous viviparous females and nymphs of Mesocallis (Mesocallis) platycaryae sp. nov.

Characters	Apterous viviparous female (holotype)	Apterous viviparous female (paratype)	3^rd^ nymph (*n* = 1)	4^th^ nymph (*n* = 1)
Body length	1.120	1.010	1.020	1.030
Body width	0.470	0.450	0.490	0.490
Antenna	0.532	0.470	0.465	0.483
Antennal segment I	0.047	0.040	0.045	0.042
Antennal segment II	0.037	0.035	0.037	0.042
Antennal segment III	0.183	0.166	0.151	0.144
Antennal segment IV	0.106	0.092	0.087	0.097
Base of antennal segment V	0.084	0.077	0.082	0.089
Processus terminalis	0.074	0.062	0.064	0.069
Ultimate rostral segment	0.062	0.062	0.062	0.062
Hind femur	0.260	0.230	0.225	0.218
Hind tibia	0.396	0.361	0.342	0.302
Second hind tarsal segment	0.084	0.079	0.079	0.077
Siphunculus	0.037	0.037	0.027	0.030
Basal width of siphunculus	0.054	0.050	0.050	0.054
Distal width of siphunculus	0.032	0.035	0.032	0.030
Cauda	0.099	0.099	–	–
Basal width of cauda	0.089	0.094	–	–
Basal diameter of antennal segment III	0.015	0.012	0.012	0.015
Widest width of hind femur	0.052	0.050	0.050	0.050
Width of hind tibia at mid length	0.020	0.020	0.027	0.025
Longest dorsal cephalic seta	0.084	0.074	0.074	0.057
Longest marginal seta on abdominal tergite I	0.079	0.114	0.059	0.040
Longest seta on abdominal tergite VIII	0.151	0.129	0.092	0.094
Longest seta on antennal segment III	0.005	0.005	0.005	0.005
Longest seta on hind tibia	0.027	0.030	0.040	0.030

#### Mounted specimens.

Body dorsum pale; antennae, legs, cauda, anal plate, and genital plate pale, apex of rostrum brown (Fig. 13). Dorsal body setae thick long and dark brown, with elevated bases, sparsely spinulose shafts on part of length and large round knobs at apices (Figs 4–8, 13, 20–22, 24).

**Figures 1–12.**
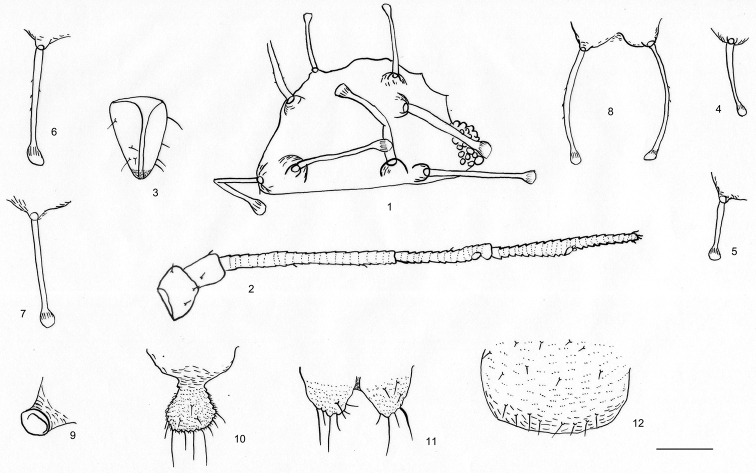
*Mesocallis platycaryae* sp. nov. Apterous viviparous female **1** dorsal view of head **2** antennal segments I–V **3** ultimate rostral segment **4** frontal seta **5** marginal seta on abdominal tergite I **6** spinal seta on abdominal tergite III **7** marginal seta on abdominal tergite III **8** spinal seta on abdominal tergite VIII **9** siphunculus **10** cauda **11** anal plate **12** subgenital plate. Scale bars: 0.05 mm.

***Head*.** Frons convex (Figs 1, 17). Dorsal setae on head similar to dorsal body setae. Head with one pair of frontal setae (Figs 4, 16, 17), one pair of setae between antennae and two pairs of posterior marginal setae between eyes (Figs 1, 16, 17). Frontal setae 5.67–6.00× as long as basal diameter of antennal segment III. Eyes with relatively few facets (Figs 1, 16). Antennae 5-segmented, 0.29–0.33× as long as body (Figs 2, 18), segments III–V with spinulose transverse imbrication; processus terminalis 0.81–0.88× as long as the base of the segment. Antennal setae very few short and pointed; segments I–V each with 2, 2, 2 or 3, 2, 1 setae, respectively; processus terminalis with four apical setae. Length of setae on segment III 0.33–0.40× as long as basal diameter of the segment. Primary rhinaria not ciliated (Figs 2, 18). Rostrum (Figs 3, 17) reaching back to between fore and mid-coxae; ultimate rostral segment thick wedge-shaped, 1.39–1.56× as long as its basal width, 0.74–0.78× as long as second hind tarsal segment, with two accessory setae

**Figures 13–15.**
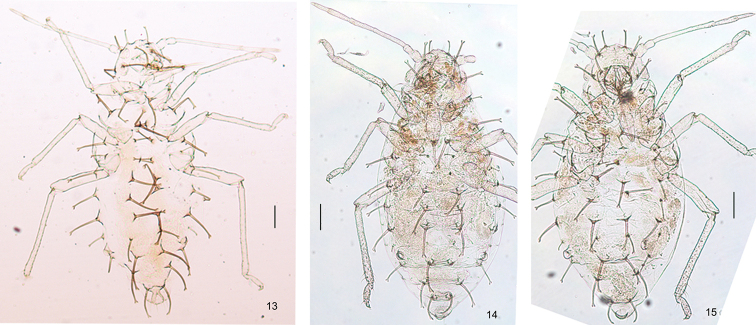
*Mesocallis platycaryae* sp. nov. Dorsal view of body **13** apterous viviparous female **14** 4^th^ nymph **15** 3^rd^ nymph. Scale bars: 0.1 mm.

***Thorax*** (Fig. 13). Pronotum with 1 pair of short, pale brown, anterior spinal setae and 2 pairs of thick, long, dark brown marginal setae; meso- and metanotum each with one pair of spinal and one pair of marginal thick, long, dark brown setae. Mesosternal furca separated (Fig. 5). Femur and trochanter partially fused; hind femur and trochanter 4.66–5.00× as long as greatest width of segment; 1.42–1.48× as long as antennal segment III. Distal half of tibiae and tarsi with spinulose transverse striae (Fig. 19); hind tibia 0.35–0.36× as long as body. Setae on legs fine, pointed; tibial distal setae similar to other tibial setae; length of setae on hind tibiae 1.38–1.50× as long as middle diameter of segment. First tarsal segments each with five ventral setae, and without dorsal setae. Second hind tarsal segment 1.13–1.28× as long as processus terminalis, 0.79–0.86× as long as antennal segment IV. Empodial setae flabellate.

***Abdomen*.** Abdominal tergites I–VII each with a single pair of spinal and a pair of marginal setae (Figs 5–7, 13, 20–22), marginal setae on tergites I and V distinctly shorter than ones on tergites II–IV (Figs 20, 21), setae on tergite VII very much shorter and pointed, not elevated at base; sometimes spinal setae on tergites III and V slightly shorter than other spinal setae; tergite VIII with one pair of thick long and dark brown dorsal setae (Figs 8, 24). Marginal setae on tergite I are 5.33–9.20× as long as basal diameter of antennal segment III; dorsal setae on tergite VIII 10.17–10.40× as long as basal diameter of antennal segment III. Siphunculi truncated (Figs 9, 22, 23), 0.68–0.75× as long as their basal widths, 0.38× as long as cauda. Cauda knob-shaped, with spinulose short striae (Figs 10, 24); 0.27–0.28× as long as its basal width, with 6–8 long and short pointed setae. Anal plate bilobed, with short spinulose striae (Figs 11, 24). Subgenital plate transversely oval (Fig. 12), with sparse spinulosity in transverse lines; with nine anterior setae, six to eight posterior setae. Gonapophyses fused, with eight short gonosetae.

**Figures 16–24.**
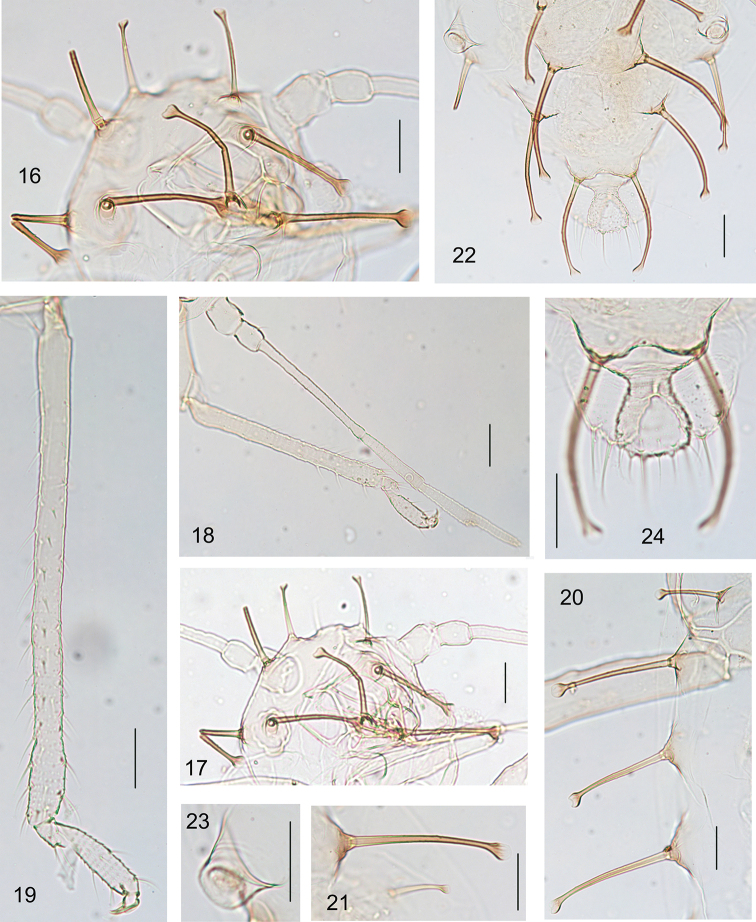
*Mesocallis platycaryae* sp. nov. Apterous viviparous female **16** dorsal view of body, dorsal setae shown **17** dorsal view of head, antennal segments I–II, and ultimate rostral segment **18** antenna, fore tibia and tarsal segment **19** hind tibia and tarsal segments **20** marginal setae on antennal segments I–IV **21** marginal setae and marginal setae on abdominal tergites IV–V **22** siphunculi, spinal and marginal setae on abdominal tergites VI–VIII, cauda and anal plate **23** siphunculus **24** dorsal setae on abdominal tergite VIII, cauda, and anal plate. Scale bars: 0.05 mm.

***Third instar nymph*.** Body oval (Fig. 14), pale brown. Cauda circular at apex, otherwise similar to apterous viviparous female.

***Fourth instar nymph*.** Body oval (Fig. 15), pale brown. Cauda circular at apex, otherwise similar to apterous viviparous female.

***Embryo*.** Dorsal body setae thick, long, and with terminal large round knobs. Head with three pairs of anterior dorsal setae, and two pairs of posterior marginal setae; pronotum with two pairs of spinal setae and one pair of marginal setae, some anterior spinal setae minute; meso- and metanotum each with one pair of spinal and one pair of marginal setae; abdominal tergites I–VII each with one pair of spinal and one pair of marginal setae; among spinal setae of metanotum and tergites I, III, and V are minute, marginal setae on tergites I–III and V–VII; spinal setae on tergites III, V, and VII are displaced.

#### Specimens examined.

***Holotype***: apterous viviparous female, **China** (Anhui Province: Yuexi County, Yaoluoping Reserve, Xiaoqiling, alt. 1100 m), 19 Jul. 2007, no. 20714-1-1-1, on *Platycarya strobilacea*, coll. J.J. Yu (NZMC). ***Paratypes***: 1 apterous viviparous female, 1 third instar nymph, and 1 fourth instar nymph (NZMC), the collection data is the same as in the holotype.

#### Taxonomic notes.

Based on the following morphological characteristics in apterae and nymphs of dorsal body setae with round knobbed apex, 5-segmented antennae, much shorter than the body, hind tibial distal setae similar to other setae on the segment, distal part of tibiae and tarsi spinulose, and abdominal tergites I–VII each with one pair of marginal setae, the new species should clearly be placed in *Mesocallis*. The species is characterised by the dark-brown dorsal body setae, which are placed on unsclerotized tuberculate bases, and by its colonisation of *Platycarya strobilacea* (Juglandaceae). *Mesocallis platycaryae* resembles *M. taoi* in the number of antennal segments, the ratio of antennae to body length, the length and the number of accessory setae of ultimate rostral segment etc., but apterae differ from those of *M. taoi* as follows: dorsal body setae dark brown, not arising from sclerites (*M. taoi* has the dorsal body setae pale but on pigmented sclerites); shafts of dorsal body setae largely smooth, only sparsely spinulose on part of length (vs long dorsal body setae with spinulose shafts); antennae and tarsi pale (vs distal part of antennal segments III–V and tarsi brown). The new species differs from *M. carpinicola* and *M. pteleae* in: ultimate rostral segment 0.06 mm long, and with two accessory setae (*M. carpinicola* and *M. pteleae*: 0.10–0.14 mm long, with four or more accessory setae), head vertex and antennal segments I–III pale (vs blackish), cauda with 6–8 setae (vs 7–15 setae). In addition, the new species may be distinguished from *M. obtusirostris* by: antennae 0.46–0.48× as long as body (*M. obtusirostris*: antennae 0.61–0.75× as long as body), ultimate rostral segment 0.74–0.78× as long as second hind tarsal segment (vs 0.50–0.55×). The difference between the new species and other species of subgenus Mesocallis may be found in the key below.

#### Distribution.

China (Anhui).

#### Host plant.

*Platycarya strobilacea* (Juglandaceae).

#### Biology.

The species lives scattered on the underside of leaves of host plant.

### Mesocallis (Mesocallis) pteleae

Taxon classificationAnimaliaHemipteraAphididae

Matsumura, 1919

19BED0FC-C2ED-5F4B-91EA-81CBCED8335B

Mesocallis pteleae Matsumura 1919: 103. Higuchi 1972: 23; Quednau 2003: 20; Qiao et al. 2005: 196; Lee et al. 2018: 4.
Agrioaphis hashibamii Shinji 1935: 287; Tseng and Tao 1938: 209; Tao 1963: 57.
Myzocallis colyricola Shinji 1941: 1148.

#### Specimens examined.

Two alate viviparous females, **China**: Hebei (Wulin Mountain), 15 Jul. 1983, no. Y4357, on *Corylus mandshurica*, coll. S.B. Tian (NZMC); 1 alate viviparous female, Hebei (Wulin Mountain), 13 Sep. 1983, no. Y4354, on *Corylus heterophylla*, coll. S.B. Tian (NZMC); 1 alate viviparous female, Gansu (Yuzhong County: Xinglong Mountain, alt. 2170m), 30 Jul. 1986, no. 8556, on *Corylus heterophylla*, coll. G.X. Zhang, J.H. Li, and T.S. Zhong (NZMC); 3 alate viviparous females, Gansu (Tianshui City: Maijishan Mountain, alt. 1700 m), 24 Jul. 1985, no. 8556, on *Corylus heterophylla*, coll. G.X. Zhang and T.S. Zhong (NZMC).

#### Distribution.

China (Hebei, Sichuan, Gansu), Japan, Korea.

#### Host plants.

*Corylus heterophylla*, C. sieboldiana var. mandshurica, *Alnus cremastogyne* in China; but, in Japan, *Alnus matsumurae*, Corylus heterophylla var. thunbergii (Shinji 1935); *C. sieboldiana*, C. sieboldiana var. mandshurica (Higuchi 1972), also recorded from *Ostrya japonica* and *Carpinus* sp. (Quednau 2003), and *Betula* spp. (Holman 2009).

#### Biology.

Pale green in life; scattered on the underside of leaves of host plants.

### Mesocallis (Mesocallis) sawashibae

Taxon classificationAnimaliaHemipteraAphididae

(Matsumura, 1917)

7972E5D0-5048-5583-B6A4-EBC58C397148

Myzocallis sawashibae Matsumura 1917: 374; Matsumura 1919: 103.
Mesocallis sawashibae (Matsumura): Higuchi 1972: 24; Quednau 2003: 20; Qiao et al. 2005: 198; Lee et al. 2018: 8.
Neocallis carpinicola Matsumura 1919: 105.
Nippochaitophorus moriokaensis Takahashi 1961: 247.

#### Specimens examined.

Nine alate viviparous females, **China**: Hebei (Changli County), 30 May 1984, no. 5518, on *Corylus heterophylla*, coll. S.B. Tian (NZMC); 1 alate viviparous female, Hebei (Wulin Mountain), 15 Sep. 1983, no. Y4357, on *Corylus mandshurica*, coll. S.B.Tian (NZMC).

#### Distribution.

China (Hebei), Japan, Korea.

#### Host plants.

*Corylus heterophylla* and *C. mandshurica* in China; in Japan, *Corpinus cordata* (Higuchi 1972), *Corpinus erosa*, *C. coreana* (Quednau 2003).

#### Biology.

White in life; scattered on the underside of leaves of host plants.

### Mesocallis (Mesocallis) taoi

Taxon classificationAnimaliaHemipteraAphididae

Quednau, 2003

E69A8036-E8BA-50C4-BA8D-62A32AB5277E

Mesocallis taoi Quednau 2003: 20, 53.

#### Distribution.

China (Sichuan).

#### Host plants.

*Alnus cremastogyne*.

### Mesocallis (Paratinocallis) yunnanensis

Taxon classificationAnimaliaHemipteraAphididae

(Zhang, 1985)

B82E93AA-93FE-5258-B9F0-42CE8166000B

Paratinocallis yunnanensis Zhang 1985: 220; Qiao et al. 2005: 211.
Mesocallis (Paratinocallis) yunnanensis (Zhang): Quednau 2003: 21.

#### Specimens examined.

Three alate viviparous females, **China**: Yunnan (Lijiang City: Yulongxueshan Mountain), 30 May 1984, no. 7192, on *Corylus heterophylla*, coll. T.S. Zhong (NZMC).

#### Distribution.

China (Yunnan).

#### Host plants.

*Corylus heterophylla*.

#### Biology.

Beige in life; infesting the underside of leaves of host plants.

##### Key to the species of *Mesocallis* in China

**Table:** 

1	Abdominal tergites I–IV each with one pair of marginal setae	**2**
–	Abdominal tergites I–IV each with two or three pairs of marginal setae	**6**
2	Dorsal body setae dark brown; shafts of setae mainly smooth, only sparsely spinulose for part of length; on *Platycarya strobilacea* (Juglandaceae)	***M. platycaryae* sp. nov.**
–	Dorsal body setae unpigmented; with spinulose shafts; on plants of Betulaceae	**3**
3	In the alatae anterior part of head black; antennal segment III black; ultimate rostral segment 0.7–1.4× as long as hind second tarsal segment	**4**
–	In the alatae anterior part of head pale; antennal segment III black in whole, or dorsal half, or only apex; ultimate rostral segment 0.6–0.9× as long as hind second tarsal segment	**5**
4	In alatae: processus terminalis 0.6–0.8× as long as the base of the segment; ultimate rostral segment 0.7–0.9× as long as hind second tarsal segment; first tarsal segments with five ventral setae; in apterae and nymph: marginal setae of abdominal tergites V and VII minute or very short	***M. taoi*** ^1^
–	In alatae: processus terminalis 0.9–1.2× as long as the base of the segment; ultimate rostral segment 1.2–1.4× as long as hind second tarsal segment; first tarsal segments with six ventral setae; in alatoid nymph: marginal setae of abdominal tergites V and VII slightly shortened than those on other tergites	***M. pteleae***
5	Some abdominal tergites with duplicated spinal setae and/or with an intercalary seta developed amidst spinal setae; processus terminalis 0.6–0.7 × as long as the base of the segment; in alatae antennal segment III black except for its very base, antennal segment IV sometimes with secondary rhinaria	***M. alnicola***
–	Abdominal tergites each with one pair of spinal setae; processus terminalis 1.0–1.2× as long as the base of the segment; in alatae antennal segment III pale with apex, antennal segment IV without secondary rhinaria	***M. sawashibae***
6	In alatae: processus terminalis 0.9–1.0× as long as the base of the segment; antennal segment IV with secondary rhinaria; ultimate rostral segment 0.9–1.0× as long as hind second tarsal segment	***M. corylicola***
–	In alatae: processus terminalis 0.6–0.7× as long as the base of the segment; antennal segment IV without secondary rhinaria; ultimate rostral segment 1.5–1.7× hind second tarsal segment	***M. yunnanensis***

## Supplementary Material

XML Treatment for Mesocallis

XML Treatment for Mesocallis (Mesocallis) alnicola

XML Treatment for Mesocallis (Paratinocallis) corylicola

XML Treatment for Mesocallis (Mesocallis) platycaryae

XML Treatment for Mesocallis (Mesocallis) pteleae

XML Treatment for Mesocallis (Mesocallis) sawashibae

XML Treatment for Mesocallis (Mesocallis) taoi

XML Treatment for Mesocallis (Paratinocallis) yunnanensis

## Footnotes

The information on M. taoi is based on the original description by Quednau (2003).
